# Exosomes/miRNAs as mediating cell-based therapy of stroke

**DOI:** 10.3389/fncel.2014.00377

**Published:** 2014-11-10

**Authors:** Hongqi Xin, Yi Li, Michael Chopp

**Affiliations:** ^1^Department of Neurology, Henry Ford HospitalDetroit, MI, USA; ^2^Department of Physics, Oakland UniversityRochester, MI, USA

**Keywords:** cell-based therapy, multipotent mesenchymal stromal cell (MSC), Exosome, microRNAs (miRNAs), bio-information transfer, stroke

## Abstract

Cell-based therapy, e.g., multipotent mesenchymal stromal cell (MSC) treatment, shows promise for the treatment of various diseases. The strong paracrine capacity of these cells and not their differentiation capacity, is the principal mechanism of therapeutic action. MSCs robustly release exosomes, membrane vesicles (~30–100 nm) originally derived in endosomes as intraluminal vesicles, which contain various molecular constituents including proteins and RNAs from maternal cells. Contained among these constituents, are small non-coding RNA molecules, microRNAs (miRNAs), which play a key role in mediating biological function due to their prominent role in gene regulation. The release as well as the content of the MSC generated exosomes are modified by environmental conditions. Via exosomes, MSCs transfer their therapeutic factors, especially miRNAs, to recipient cells, and therein alter gene expression and thereby promote therapeutic response. The present review focuses on the paracrine mechanism of MSC exosomes, and the regulation and transfer of exosome content, especially the packaging and transfer of miRNAs which enhance tissue repair and functional recovery. Perspectives on the developing role of MSC mediated transfer of exosomes as a therapeutic approach will also be discussed.

## Introduction

The therapeutic effects of cell-based therapy, such as for the treatment of stroke, with multipotent mesenchymal stromal cells (MSCs) have demonstrated particular promise. Systemic administration of MSCs as a treatment for stroke (Chen et al., 2001a,b; Li et al., 2001; Chopp and Li, 2002; Hessvik et al., 2013), has demonstrated that MSCs promote central nervous system (CNS) plasticity and neurovascular remodeling which lead to functional benefit (Caplan and Dennis, 2006; Zhang et al., 2006b; Chopp et al., 2008; Dharmasaroja, 2009; Li and Chopp, 2009; Zhang and Chopp, 2009, 2013; Borlongan et al., 2011; Herberts et al., 2011). Instead of the replacement of damaged cells, cell-based therapy provides therapeutic benefit by remodeling of the CNS, i.e., by promoting neuroplasticity, angiogenesis and immunomodulation (Chen et al., 2001b; Chopp and Li, 2002; Chopp et al., 2008; Li and Chopp, 2009; Zhang and Chopp, 2013; Liang et al., 2014). Early studies posited that the therapeutic efficacy of transplanted MSCs was attributed to their subsequent differentiation into parenchymal cells which repairs and replaces damaged tissues. However, studies in animal models and patients demonstrated that only a very small number of transplanted MSCs localize to the damage site and surrounding area, while most of the MSCs were localized in the liver, spleen and lungs (Phinney and Prockop, 2007). In addition, apparent evidence of MSC differentiation likely resulted from the fusion of transplanted MSCs with endogenous cells (Spees et al., 2003; Vassilopoulos et al., 2003; Konig et al., 2005; Ferrand et al., 2011). Supported by robust data, our present understanding of how MSCs promote neurological recovery is through their interaction with brain parenchymal cells. MSCs produce and induce within parenchymal cells biological effectors, e.g., neurotrophic factors, proteases, and morphogens, which subsequently enhance the neurovascular microenvironment surrounding the damaged area, as well as remodel remote tissue (Chen et al., 2002; Lu et al., 2002; Mahmood et al., 2004; Gao et al., 2005, 2008; Xin et al., 2006, 2010, 2011, 2013a; Zhang et al., 2006c, 2009; Qu et al., 2007; Zacharek et al., 2007; Shen et al., 2008, 2010, 2011b; Xu et al., 2010; Hermann and Chopp, 2012; Ding et al., 2013; Zhang and Chopp, 2013). Though the mechanisms which underlie the interaction and communication between the exogenously administered cells, e.g., MSCs, and brain parenchymal cells are not fully understood, the paracrine effect hypothesis has been strengthened by recent evidence that stem cells release extracellular vesicles which elicit similar biological activity to the stem cells themselves (Lai et al., 2011; Camussi et al., 2013; Xin et al., 2013b). These released extracellular lipid vesicles, provide a novel means of intercellular communication (Raposo and Stoorvogel, 2013; Fujita et al., 2014; Record et al., 2014; Turturici et al., 2014; Zhang and Grizzle, 2014). A particularly important class of extracellular vesicles released by stem cells and MSCs, is exosomes, and accumulating data show that MSCs release large amounts of exosomes which mediate the communication of MSCs with other cells (Collino et al., 2010; Hass and Otte, 2012; He et al., 2012; Xin et al., 2012; Lee et al., 2013; Roccaro et al., 2013; Wang et al., 2014). Here, we focus our discussion on exosomes derived from MSCs, the biogenesis of MSC exosomes, cargo packaging (especially the miRNAs) and intercellular communication, and discuss new opportunities in modifying exosomal cargo to develop exosome-based cell-free therapeutics.

### Characteristic of exosomes

Lipid vesicles can be released by various types of cells, and they have been found in the supernatants from a wide variety of cells in culture, as well as in all bodily fluids (Yang et al., 2014; Yellon and Davidson, 2014; Zhang and Grizzle, 2014). The shedding of microvesicles and exosomes is likely a general property of most cells. Initial studies on cell released vesicles were reported in the 1960s (Roth and Luse, 1964; Schrier et al., 1971; Dalton, 1975), and the most common term, exosome, as applied to cell-derived vesicles was first defined by Trams et al. (1981); since they believe that these “exfoliated membrane vesicles may serve a physiologic function” and “it is proposed that they be referred to as exosomes” (Trams et al., 1981), (1, nomenclature).

Box 1 **Nomenclature.**

Currently, the use of the term ‘exosomes’ for MVB-derived extracellular vesicles (EVs) is widely accepted in the field; however, the large variety of EVs secreted by cells and the technical difficult to definitively discriminate small EVs from exosomes in the culture media using currently available methods has led to the less stringent usage of the term, exosomes. Exosomes are presently characterized as either small EVs (of 30–100 nm diameter) measured by transmission electron microscopy (TEM)), or as EVs recovered after 100000g ultracentrifugation. As Gould and Raposo proposed recently, given the absence of perfect identification of EVs′ of endosomal origin, researchers are recommended to explicitly state their use of terms, choose their terms based on precedent and logical argument, and apply them consistently throughout a piece of work (Gould and Raposo, 2013). Since the EVs identified and employed in our studies fulfill the above mentioned two characteristics (i.e.,TEM and 100000g untracentrifugation), therefore, exosomes are likely the primary constituents of the EVs. Here, in this manuscript, we use the term ‘exosomes’ as defined by Trams et al. (Trams et al., 1981), however, we do not exclude the possibility of other non-exosomal microvesicle components within the content of our injected precipitate, and we do not exclude a contribution of non-exosomal microvesicles to mediating stroke recovery.

**Figure 1:**
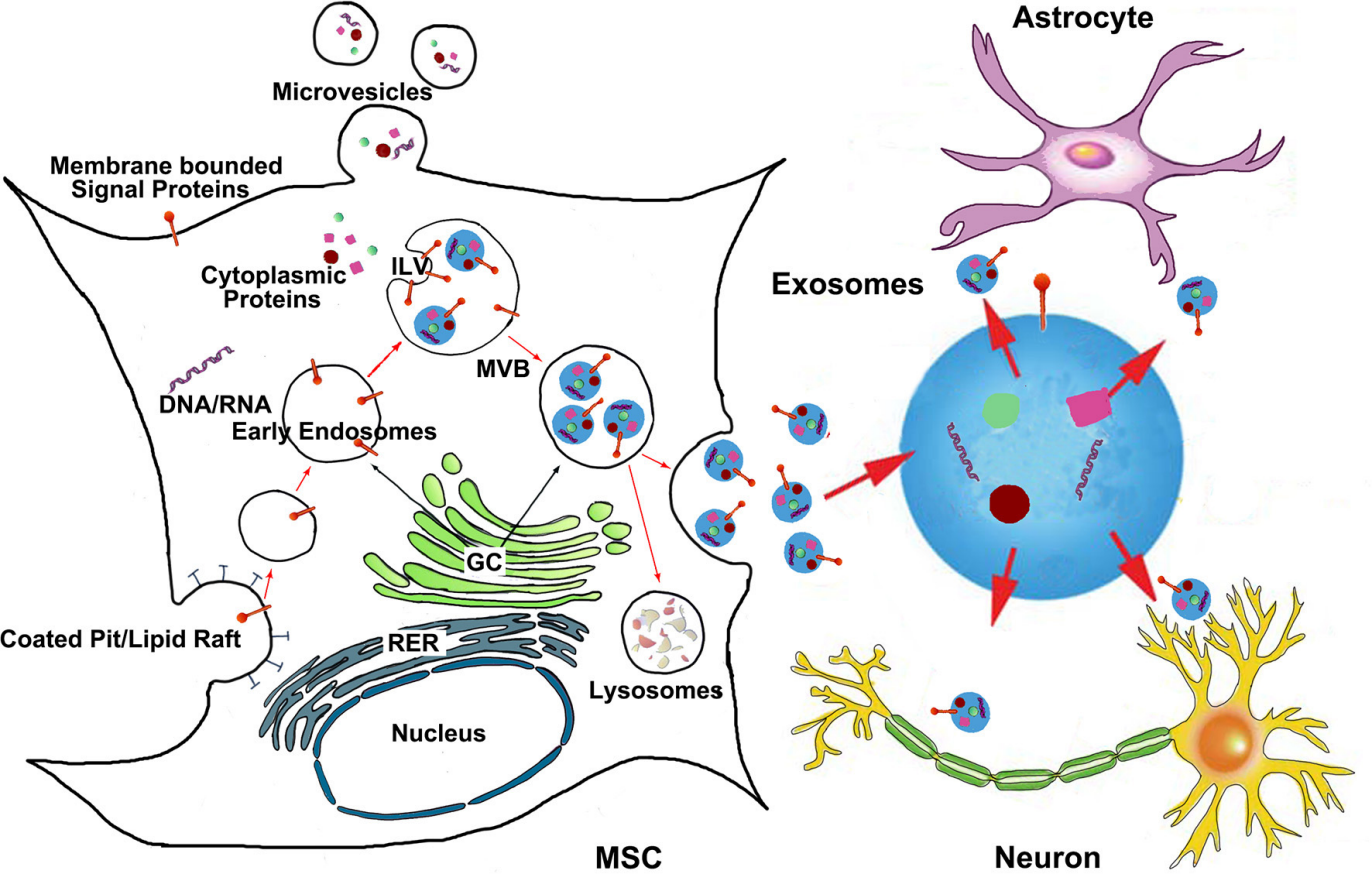
**The generation of MSC exosomes and bio-information shuttling between MSCs and brain parenchymal cells via exosomes**. Exosomes are generated in the late endosomal compartment by inward budding of the limiting membrane of MVB. The exosome-filled MVBs are either fused with the plasma membrane to release exosomes or sent to lysosomes for degradation. Microvesicles are plasma membrane derived particles that are released into the extracellular environment by the direct outward budding and fission of the plasma membrane. The bio-information carried by MSC exosomes then transfer to brain parenchymal cells like astrocytes and neurons. ILV, intraluminal vesicles; MVB, multivesicular body; GC, Golgi complex; RER, rough endoplasmic reticulum.

Extracellular released vesicles mainly include exosomes and microvesicles (Momen-Heravi et al., 2013). Exosomes are endocytic origin small-membrane vesicles. Eukaryotic cells periodically engulf small amounts of intracellular fluid in the specific membrane area, forming a small intracellular body called endosome (Thery et al., 2002). The early endosome matures and develops into the late endosome, during the maturation process, the inward budding of the endosomal membrane forms the intraluminal vesicles (ILV) which range in size from approximately 30–100 nm in diameter. The late endosome containing ILVs is also referred to as, a multivesicular body (MVB) and proteins are directly sorted to the MVBs from rough endoplasmic reticulum and Golgi complex (Thery et al., 1999), as are mRNAs, microRNAs, and DNAs (Villarroya-Beltri et al., 2013). The MVBs may either fuse with the lysosome and degrade their contents or fuse with the plasma membrane of the cell, releasing their ILVs to the extracellular environment (Figure 1). These vesicles are then referred as exosomes (Van Niel et al., 2006). Microvesicles are small, plasma membrane derived particles that are released into the extracellular environment by the outward budding and fission of the plasma membrane (Amano et al., 2001; Cocucci et al., 2009; Muralidharan-Chari et al., 2010). Unlike the large size of microvesicle (100~1000 nm in diameter), exosomes have a smaller size, ~30–100 nm in diameter (Stoorvogel et al., 2002). Exosome density in sucrose is located at 1.13–1.19 g/ml, and exosomes can be collected by ultracentrifugation at 100,000 g (Thery et al., 2006). The exosome membranes are enriched with cholesterol, sphingomyelin, and ceramide which are contained in lipid rafts (Thery et al., 2006). Most exosomes contain conserved proteins such as tetraspanins (CD81, CD63, and CD9), Alix and Tsg101, as well as the unique tissue/cell type specific proteins that reflect their cellular source. A precise and clear distinction between these vesicles (exosomes and microvesicles) is still lacking, and it is technically difficult to definitively separate them from the culture media by currently available methods like ultracentrifugation, density gradient separation, chromatography and immunoaffinity capture methods (Corrado et al., 2013). Exosomes are released by most cell types under physiological conditions. The amount of exosomes released from MSCs is highly related to cellular proliferation rate, and the exosome production is inversely correlated to the developmental maturity of the MSCs (Chen et al., 2013b). The release of extracellular vesicles can be altered by cellular stress and damage (Hugel et al., 2005; Greenwalt, 2006). Increased release of extracellular vesicles is associated with the acute and active phases of several neurological disorders (Hugel et al., 2005; Horstman et al., 2007). The distinctions between exosomes and other extracellular vesicles (such as microvesicles) are beyond the scope of this review and will not be discussed in detail here.

### MSCs robustly release exosomes

Human MSC conditioned medium can reduce myocardial infarct size in patients with acute myocardial infarction (Timmers et al., 2007), and Reduction of myocardial infarct size by human mesenchymal stem cell conditioned medium, probably by increasing myocardial perfusion (Timmers et al., 2011). These therapeutic effects were then subsequently attributed to MSC derived exosomes (Lai et al., 2010). Thereafter, MSC exosomes were widely observed and tested in several disease models (Lee et al., 2012; Reis et al., 2012; Xin et al., 2012; Li et al., 2013; Tomasoni et al., 2013; Sdrimas and Kourembanas, 2014; Tan et al., 2014; Zhu et al., 2014).

Compared to other cells, MSCs can produce large amounts of exosomes (Yeo et al., 2013). There are no differences in terms of morphological features, isolation and storage conditions between exosomes derived from MSCs and other sources (Yeo et al., 2013). The MSC is the most prolific exosome producer when compared to other cell types known to produce exosomes (Yeo et al., 2013). By transfecting human ESC-derived mesenchymal stem cells (hESC-MSCs) with a lentivirus carrying *myc* gene, Chen et al. generated an immortalized hESC-MSCs cell line. Exosomes from MYC-transformed MSCs were able to reduce relative infarct size in a mouse model of myocardial ischemia/reperfusion injury. They found that MYC transformation may be a practical strategy in ensuring an infinite supply of cells for the production of exosomes in the milligram range as either therapeutic agents or delivery vehicles. Additionally, the increased proliferative rate by MYC transformation reduces the time for cell production and thereby reduces production costs. Chen et al. (2011), thus, making MSCs an efficient and effective “factory” for mass production of exosomes.

### The cargo of MSC exosomes

Exosomes are complex “living” structures generated by many cell types containing a multitude of cell surface receptors (Shen et al., 2011a; Yang and Gould, 2013), encapsulating proteins, trophic factors, miRNAs, and RNAs (Koh et al., 2010; Lai et al., 2011, 2012, 2013b; Record et al., 2011; Xin et al., 2012; Chen and Lim, 2013; Katakowski et al., 2013; Tomasoni et al., 2013; Yeo et al., 2013). These bioactive molecules can mediate exosomal intercellular communication (Zhang and Grizzle, 2014; Zhang and Wrana, 2014).

The exosome cargo is dependent on the cell type of origin (Raposo and Stoorvogel, 2013). Besides the common surface markers of exosomes, such as CD9 and CD81, MSCs contain specific membrane adhesive molecules, including CD29, CD44, and CD73 that are expressed on the MSC generated exosomes (Lai et al., 2012). Further, the specific conditions of cell preparation affect the exosome cargo (Kim et al., 2005; Park et al., 2010). In the MSC derived exosome, protein components also changed when exosomes were obtained from different MSC cultured media. In their study, Lai et al. found that 379, 432, and 420 unique proteins, detected by means of liquid chromatography-mass spectrometry/mass spectrometry in three independent batches of MSC derived exosomes, and only 154 common proteins are present (Lai et al., 2012). In addition to the protein cargo, RNAs, e.g., messenger RNA (mRNA) and miRNAs are encapsulated in MSC exosomes. MiRNAs encapsulated in MSC-derived microparticles are predominantly in their precursor form (Chen et al., 2010). However, other studies have demonstrated that various miRNAs are present in MSC exosomes, and the miRNA cargo participates in the cell-cell communication to alter the fate of recipient cells (Koh et al., 2010; Xin et al., 2012, 2013c; Katakowski et al., 2013; Lee et al., 2013; Ono et al., 2014).

Environmental challenges, such as activation or stress conditions, influence the composition, biogenesis, and secretion of exosomes. Possibly, exosome secretion is an efficient adaptive mechanism that cells modulate intracellular stress situations and modify the surrounding environment via the secretion of exosomes. By preconditioning (Yu et al., 2013) or genetic manipulation (Kim et al., 2007b) of dendritic cells, the exosome secretion profile of these cells can be modified. The proteomic profiles of adipocyte-derived exosomes have been characterized (Sano et al., 2014). The authors found that protein content of the exosomes produced from cultured 3T3-L1 adipocytes was changed when they exposed the cells to hypoxic conditions. Quantitative proteomic analysis showed that 231 proteins were identified in the adipocyte-derived exosomes, and the expression levels of some proteins were altered under hypoxic conditions. The total amount of proteins in exosomes increased by 3-4-fold under hypoxic conditions (Sano et al., 2014). Another study found that the miRNA content of dendritic cell exosomes was affected by the maturation of the cells (Montecalvo et al., 2012), and similarly, compared with those from control cells, exosomes from mast cells contain different mRNAs when the cells were exposed to oxidative stress (Eldh et al., 2010). Furthermore, stressed cells that released exosomes conferred resistance against oxidative stress to recipient cells (Eldh et al., 2010), suggesting that cells modulate intracellular stress situations and modify the surrounding environment via the secretion of exosomes. The MSC exosome profile can be modified by pretreatment, as well. When MSCs were *in vitro* exposed to brain tissue extracted from rats subjected to middle cerebral artery occlusion (MCAo), the miR-133b levels in MSCs and their released exosomes were significantly increased compared to MSCs exposed to normal rat brain tissue extracts (Xin et al., 2012), indicating that MSCs used for stroke treatment will modify their gene expression and subsequently affect their exosome cargo. Thus, there is a feedback between the MSC and its environment, and through which ischemic conditions will modify the exosome contents, and consequently, the secreted exosomes affect and modify the tissue environment. Though we only tested one specific miRNA in our study, it is reasonable to propose that other miRNAs or other cargos of MSC exosome were modified by the post ischemic condition. i.e., other groups also demonstrated that miR-22 in MSC exosomes were enriched following ischemic preconditioning (Feng et al., 2014).

### MSC derived exosomes transfer bio-information to recipient cells via miRNA

MiRNAs are non-protein coding, short ribonucleic acid (usually 18–25 nucleotides) molecules found in eukaryotic cells. Via binding to complementary sequences on target mRNA transcripts, miRNAs post-transcriptionally control gene expression (Bartel, 2004, 2009). MiRNAs constitute a major regulatory gene family in eukaryotic cells (Bartel, 2004; Zhang et al., 2006a, 2007; Fiore et al., 2008). MiRNAs are master molecular switches, concurrently affecting translation of, possibly, hundreds of mRNAs (Cai et al., 2009; Agnati et al., 2010). Over 1000 miRNAs are encoded by the human genome (Bartel, 2004) and they target about 60% of mammalian genes (Lewis et al., 2005; Friedman et al., 2009), and are abundant in many human cell types (Lim et al., 2003). By affecting gene expression, miRNAs are likely involved in most biological processes (Brennecke et al., 2003; Chen et al., 2004; Cuellar and McManus, 2005; Harfe et al., 2005; Lim et al., 2005). Based on the master gene regulation role of miRNAs, though MSC exosomes have the potential for protein cargo transfer (Zhang et al., 2014), we envisage that compared with the delivery of proteins, transfer of miRNA may have dramatic effects on the network of proteins and RNAs of the recipient cells.

Exosomes are well suited for small functional molecule delivery (Zomer et al., 2010). Increasing evidence indicates that they play a pivotal role in cell-to-cell communication (Mathivanan et al., 2010) and act as biological transporters (Denzer et al., 2000; Fevrier and Raposo, 2004; Lotvall and Valadi, 2007; Smalheiser, 2007; Valadi et al., 2007; Mathivanan et al., 2010; Lee et al., 2011; Record et al., 2011; Von Bartheld and Altick, 2011; Mittelbrunn and Sanchez-Madrid, 2012; Boon and Vickers, 2013; Raposo and Stoorvogel, 2013). Importantly, by being encapsulated and contained within the exosomes, the RNA is protected from the digestion of RNAase or trypsin (Valadi et al., 2007). Multiple studies show that exosomes transfer miRNAs to recipient cells (Valadi et al., 2007; Hergenreider et al., 2012). The transferred miRNAs then modify the recipient cell's characteristics. Shimbo et al. introduced synthetic miR-143 into cells, and the miR-143 was enveloped in released exosomes (Shimbo et al., 2014). The secreted exosome-formed miR-143 is transferred to osteosarcoma cells and subsequently significantly reduced the migration of osteosarcoma cells (Shimbo et al., 2014). Recent studies show that MSC exosomes regulate recipient cell protein expression and modify cell characteristics through the miRNA transfer (Xin et al., 2012; Lee et al., 2013; Wang et al., 2014). Exosomal transfer of miR-23b from the bone marrow may promote breast cancer cell dormancy in a metastatic niche (Ono et al., 2014). The master gene regulation role of miRNAs encapsulated within exosomes, determines their major role in the modification of recipient cells.

### Exosomes shuttle miRNAs as regulators for stroke recovery after MSC therapy

In the nervous system, exosomes mediate cell-cell communication including the transfer of synaptic proteins, mRNAs and microRNAs (Smalheiser, 2007). The role of miRNAs at various stages of neuronal development and maturation has been recently elucidated (Costa-Mattioli et al., 2009; Saba and Schratt, 2010; Olde Loohuis et al., 2012). Numerous miRNAs are expressed in spatially and temporally controlled manners in the nervous system (Kapsimali et al., 2007; Bak et al., 2008; Dogini et al., 2008; Kocerha et al., 2009; Sethi and Lukiw, 2009; Ziu et al., 2011), suggesting that miRNAs have important functions in the gene regulatory networks involved in adult neural plasticity (Sethi and Lukiw, 2009; Liu and Xu, 2011; Mor et al., 2011; Goldie and Cairns, 2012). Stroke induces changes in the miRNA profile of MSCs and within their released exosomes (Jeyaseelan et al., 2008; Lusardi et al., 2014), and miRNAs actively participate in the recovery process after stroke (Liu et al., 2013).

MiR-133b promotes functional recovery in Parkinson's disease (Kim et al., 2007a) and appears essential for neurite outgrowth and functional recovery after spinal cord injury in adult zebra-fish (Yu et al., 2011). Moreover, miR-133b regulates the expression of its targets, connective tissue growth factor (CTGF), a major inhibitor of axonal growth at injury sites in the CNS in mammals (White and Jakeman, 2008; Duisters et al., 2009) and down-regulates Ras homolog gene family, member A (RhoA) protein expression (Care et al., 2007; Chiba et al., 2009). In our series of studies, we first found that miR-133b is substantially down-regulated in rat brain after MCAo, and MSC administration significantly increased the miR-133b level in the ischemic cerebral tissue. When MSCs were exposed to ischemic brain extracts, the miR-133b level was increased in exosomes released from these MSCs. We then treated primary cultured neurons and astrocytes with these exosomes, and found the miR-133b level in the neurons and astrocytes were increased, suggesting that the exosomes mediate the miR-133b transfer from MSCs to the neurons and astrocytes. Further *in vitro* knockdown of miR-133b in MSCs directly confirmed that the increased miR-133b level in astrocytes is attributed to their transfer from MSCs to neural cells, and exosomal miR-133b from MSCs significantly increased the neurite branch number and total neurite length (Xin et al., 2012). Compared with administration of normal MSCs, *in vivo* administration of MSCs with increased or decreased miR-133b (MSCs modified using lentivirus with miR-133b knocked-in or knocked-down) to rats subjected to MCAo resulted in promotion or inhibition of neurite outgrowth, respectively (Xin et al., 2012). Correspondingly, *in vitro* and *in vivo*, we also observed the transfer of miR-133b from MSCs to astrocytes via exosomes down-regulated CTGF expression, which may thin the glial scar and benefit neurite outgrowth. In contrast, treatment of stroke in rats with MSCs containing increased miR-133b, inhibited RhoA expression in neurons which enhanced the regrowth of the corticospinal tract after injury (Dergham et al., 2002; Holtje et al., 2009). Down-regulation of CTGF and RhoA by miR-133b stimulated neurite outgrowth and thereby improved functional recovery after stroke (Xin et al., 2012). This proof-of-concept study, provides the first demonstration that MSCs communicate with astrocytes and neurons and regulate neurite outgrowth by transfer of miRNAs (miR-133b) via exosomes. The identification of exosomes released from MSCs as a shuttle that carries miR-133b to astrocytes and neurons after cerebral ischemia helps to explain, at least in-part, how the exogenous MSCs contribute to neurological recovery after stroke. Exosome delivery of functional miRNAs, e.g., miR-133b, that promote neurite outgrowth may show benefit in other neurological diseases, in addition to stroke.

### Exosomes as an alternative therapeutic candidate of MSCs on stroke

MSC exosomes serve as a vehicle to transfer protein, mRNA, and miRNA to distant recipient cells, altering the gene expression of the recipient cells. Recently, MSC exosomes have been found to be efficacious in an increasing number of animal models for the treatment of diseases such as liver fibrosis (Li et al., 2013), liver injury (Tan et al., 2014), hypoxic pulmonary hypertension (Lee et al., 2012), acute lung injury (Sdrimas and Kourembanas, 2014; Zhu et al., 2014), acute kidney injury (Gatti et al., 2011; Reis et al., 2012; Tomasoni et al., 2013), and cardiovascular diseases (Lai et al., 2011). We demonstrated that systemic treatment of stroke with cell-free exosomes derived from MSCs significantly improve neurological outcome and contribute to neurovascular remodeling (Xin et al., 2013b). This approach is the first to consider treatment of stroke solely with exosomes.

Development of gene therapy vehicles for diffuse delivery to the brain is one of the major challenges for clinical gene therapy. By using miRNA mimics or antagonists, miRNA-based strategies have recently emerged as a promising therapeutic approach for specific diseases. However, despite its exciting potential, the bottleneck of this approach is delivery of miRNA; an optimal delivery system must be found before their clinical application. Researchers developed a number of miRNA delivery systems (Zhang et al., 2013), including liposomes (Lv et al., 2006), and peptide transduction domain–double-stranded RNA-binding domain (Eguchi and Dowdy, 2009). However, synthetic materials which are employed in the above systems, limited their use. Thus, the advantages of exosomes as delivery systems are apparent; they only contain biogenic substances and are readily transferred into target cells, as well as they have potentially wide utility for the delivery of nucleic acids, and possibly for selectively targeting cells. We and others have shown that MSCs can act as “factories” for the generation of exosomes, and that the cargo within these exosomes, including the miRNAs, may be regulated by altering the genetic character of the MSCs, e.g., by transfecting the MSCs with specific genes (Zomer et al., 2010; Bullerdiek and Flor, 2012; Hu et al., 2012; Katakowski et al., 2013; Xin et al., 2013c). We have also successfully modulated the miRNA content of the MSC generated exosomes and thereby modulated neurovascular plasticity and neurological recovery from stroke (Xin et al., 2013c). Given that MSC exosomes promote recovery (Xin et al., 2013b) and MSCs release exosomes *in vivo*, we propose that MSC generated exosomes with enhanced expression of beneficial miRNAs (e.g., miR-133b) may provide improved recovery benefits.

Another development direction for the exosome treatment of disease is the targeting of recipient cells. We demonstrate a significant therapeutic and neuroplasticity effect of systemic exosome administration (Xin et al., 2013b). Considering the nano size of exosomes, they likely enter into the brain (Lakhal and Wood, 2011). Adhesive molecules are expressed on the exosome membrane (Clayton et al., 2004), which may facilitate entry into the brain. Thus, systemic exosome administration may be a means by which to deliver the active components of cell-based therapy to the CNS. To improve exosomal targeting, we may also consider engineering and tailoring cell membrane proteins, e.g., the engineering of dendritic cells to express an exosomal membrane protein, Lamp2b, fused to the neuron-specific RVG peptide3 (Alvarez-Erviti et al., 2011). Alvarez-Erviti et al. demonstrated effective delivery of functional siRNA into mouse brain by systemic injection of exosomes, and targeted the exosomes to neurons (Alvarez-Erviti et al., 2011). These data indicate that specifically targeting neural cells is feasible by modifying exosomal membrane proteins.

## Conclusion and prospects

Exosomes derived from MSCs, carry, and transfer their cargo (e.g., miRNAs) to parenchymal cells, and thereby mediate brain plasticity, and the functional recovery from stroke. For the intricate blend of paracrine factors needed, exosomes may be ideal carriers for treatment of a complicated disease such as stroke. Specifically modifying the miRNA content of MSC generated exosomes to modulate the therapeutic response for stroke may enhance their therapeutic application.

Cell-based therapies are in clinical trials for stroke and other neurological diseases (Zhou et al., 2013) and there is a robust literature on the efficacy of cell-based therapies for stroke (Hess and Borlongan, 2008). However, there are multiple benefits in transplanting exosomes rather than in transplanting the whole “factory,” the cell, into the body. In contrast to exogenously administered cells delivered systemically, exosomes, given their nano dimension may readily enter the brain and easily pass through the blood brain barrier (BBB) (Alvarez-Erviti et al., 2011; Kooijmans et al., 2012; Anthony and Shiels, 2013; Gheldof et al., 2013; Meckes et al., 2013). Exogenously administered MSCs may have many adverse effects, i. e. tumor modulation and malignant transformation. (Herberts et al., 2011; Wong, 2011), and they may lodge and initially obstruct small vessels in organs (Gao et al., 2001; Chen et al., 2013a). Exosomes given their min size, in contrast, have no vascular obstructive effect, and have no apparent adverse effects.

One case has been reported where exosomes were used for treatment for severe acute graft vs. host disease (Kordelas et al., 2014) in which MSC exosomes did not show any side effects. Side effects of exosome therapies were also not observed in any of the tumor vaccination studies which were performed in humans (Mignot et al., 2006; Viaud et al., 2008). Prion diseases are infectious neurodegenerative disorders linked to the accumulation of the abnormally folded prion protein (PrP) scrapie (PrPsc) in the CNS. Once present, PrPsc catalyzes the conversion of naturally occurring cellular PrP (PrPc) to PrPsc. Recent studies show both PrPc and PrPsc were actively released into the extracellular environment by PrP-expressing cells before and after infection with sheep prions, respectively, and the release associated with exosomes. Even though EV administration appears safe and no side effects have been observed so far, it should be noted, that exosomes may contribute to intercellular membrane exchange and the spread of prions (Fevrier et al., 2004; Klohn et al., 2013). Since fetal calf serum is used for *in vitro* culturing MSCs and amplifying the exosomes, it may bring the risk of prion disease spreading by exosomes, but this risk may be carried by *in vitro* cultured MSCs as well. However, the risk associated with exosome therapies is rather low. Table 1 shows the pros and cons of MSCs based therapy and MSC exosomes based therapy.

**Table 1:** **Pros and Cons of MSCs based therapy and MSC exosomes based therapy**.

**MSCs BASED THERAPY**	
Pros	Living cell; continuously release exosomes or other soluble factors; potency of differentiation and replacement.
Cons	tumor modulation; malignant transformation; lodge and initially obstruct small vessels in organs.
**MSC EXOSOME BASED THERAPY**	
Pros	No vascular obstructive effect; no apparent adverse effects; nano size ensure it easily pass through BBB.
Cons	None at present

Technical issues, such as, purity of exosomes must be addressed, since the most common isolation protocol with differential centrifugation and a sucrose gradient yield a heterogeneous product (EL Andaloussi et al., 2013; Lai et al., 2013a). Methods for mass exosome isolation should also be developed to reduce costs. For the modified exosome application, the exosome product needs to be extensively characterized, in order to assess its biological function and to avoid adverse effects.

### Conflict of interest statement

The authors declare that the research was conducted in the absence of any commercial or financial relationships that could be construed as a potential conflict of interest.
